# Ultra-sensitive nanostructured electrochemical immunosensor for selective monitoring of L-phenylalanine in phenylketonuria patients

**DOI:** 10.1007/s00604-026-07836-8

**Published:** 2026-01-23

**Authors:** Rebecca L. Houston, Eric C. Y. Law, Emad L. Izake

**Affiliations:** 1 School of Chemistry and Physics, Queensland University of Technology, Brisbane, QLD Australia; 2 The Children’s Hospital at Westmead, Sydney, NSW 2145 Australia; 3 The University of Sydney, Camperdown, NSW 2050 Australia; 4 Centre for Materials Science, Queensland University of Technology (QUT), 2 George Street, Brisbane, QLD 4000 Australia

**Keywords:** Fern-like gold nanostructures, Light-driven surface modification, Electrochemical immunosensor, Phenylalanine, Phenylketonuria

## Abstract

A low cost and rapid electrochemical immunosensor for direct screening of L-phenylalanine (L-Phe) using square wave voltammetry (SWV) is presented. Fern-like gold nanostructures were electro-deposited onto a screen-printed electrode (SPE) using chronoamperometry (CA), to enhance sensitivity and surface area. A novel light-driven surface functionalisation process was used to decorate the gold nanostructured SPE with target-specific antibody fragments and develop the L-Phe-specific immunosensor. The selectivity of the sensor was confirmed against tyrosine, tryptophan and D-phenylalanine, thus indicating the sensor’s potential for use at points of care (POC) without interference from structurally related biomolecules. The sensor demonstrated high sensitivity across the full clinical range (120 µM-1200 µM) and excellent linearity within the concentration range 1 µM to 2000 µM (R²= 0.99, LOD = 0.3 µM, LOQ = 1 µM). Quantification of L-Phe in human blood dry spots (DBS) by the new sensor and SWV were in excellent agreement with mass spectroscopy (MS) measurements by 2 independent pathology labs. The sensor was successfully integrated with an ultra-compact potentiostat in a Lab-on-a-Phone assembly for L-Phe screening in DBS, thus demonstrating its potential for use at POC and at home by phenylketonuria (PKU) patients in both remote and rural areas.

**Supplementary Information:**

The online version contains supplementary material available at 10.1007/s00604-026-07836-8.

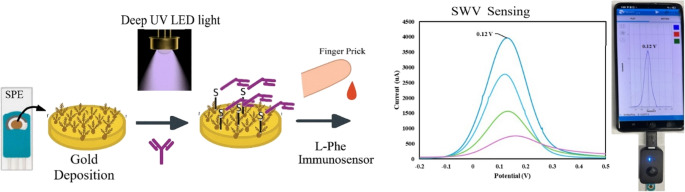

## Introduction

Phenylketonuria (PKU) is a genetic disorder that occurs to approximately 1 in 10,000 new born babies [1]. Over the past years, the global burden of PKU has become more evident with the increase in newborn screening programs [2]. Incident rates have been reported in Europe (1:850), USA (1:10,000) and China (1:15,924), underscoring the growing recognition of PKU as a worldwide health concern [3]. PKU arises from a mutation in the phenylalanine hydroxylase (PAH) enzyme during phenylalanine (Phe) metabolism. The PAH gene on chromosome 12 encodes for the conversion of L-Phe to L-tyrosine (L-Tyr), in the presence of oxygen and tetrahydrobiopterin [2]. PAH mutation occurs in various ways based on genotypes in different populations, which impacts the severity of PKU condition. This includes classic PKU, where there is little to no PAH activity, variant PKU and mild hyperphenylalaninemia (HPA), which is less severe but with some PAH functionality [3, 4]. Blood phenylalanine concentration ranging from 50 to 600 µM, 600–900 µM, 900–1200 µM and greater than 1200 µM correspond to HPA, mild PKU, moderate PKU and classic PKU conditions respectively [5].

L-Phe in PKU patients accumulates in the blood, brain and body tissue and can cause high toxicity, that results in symptoms such as eczema, seizures, intellectual disability and neurodegenerative diseases [6]. Acute phenylalanine toxicity can occur when levels exceed 1300 µmol/L, so maintaing the L-Phe concentrations below 800 µM for various organs and 500 µM for brain function is required [7]. Therfeore, there is a pressing need for highly senstive and rapid methods for montoring L-Phe concentration in PKU patients [8]. The current procedure for screening L-Phe typically require the collection of blood samples followed by Mass spectroscopy screening using LC-MS/MS assembly [11]. While LC-MS methods offer good high specificity and precision, they are time-consuming, expensive and require trained personnel for operation and data interpretation [12]. Recent research also indicated the potential for false-positive results by MS/MS methods when used for screening L-Phe in PKU patient samples [13].

Biosensors are emerging as a new alternative for sensitive, simple, low cost, and rapid screening of L-Phe [9, 10]. Various L-Phe biosensors have been demonstrated, including colorimetric, fluorescence and electrochemical sensors [11–14]. However, most electrochemical sensors detect the concentration of L-Phe indirectly through screening either by monitoring PAH activity or by requiring chemical derivatisation to generate redox-active reporter species [12, 15]. Sriram et al. recently demonstrated an electrochemical biosensor for Phe that uses bismuth telluride (Bi_2_Te_3_) as an electrocatalyst to enhance the oxidation of the amino acid and enable its detection by SWV [16]. The sensor screens the total concentration of Phe and does not differentiate between the L and D isoforms of the amino acid. Idili et al. demonstrated an aptamer-based electrochemical sensor for Phe [17]. However, the sensor suffers from interference from Tryptophan amino acid, thus leading to inaccurate determination of L-Phe in human blood.

In this work, we demonstrate a highly selective, sensitive and rapid electrochemical immunosensor for L-Phe. The new sensor was developed by depositing fern-like gold nanostructures onto SPE, thus increasing its electro-active surface area and sensitivity in electrochemical measurements. The gold nanostructures were functionalised with target-specific antibody molecules using a regent-less surface functionalisation process. LED light @280 nm was utilised to reduce the disulphide bonds within the hinge region of the antibody molecules and attach them to the sensor surface by Au-S bonds. The new immunosensor was used to screen L-Phe within the concentrations range 1 µM to 2000 µM by SWV. Positive and negative control tests confirmed the sensor’s selectivity towards L-Phe over D-Phe, tyrosine and tryptophan amino acids, thus indicating its potential for POC measurements. The sensor was utilised for the determination of L-Phe concentration in quality assurance DBS samples by SWV. The SWV results compared to those obtained by MS method at 2 independent pathology labs and excellent agreement was found between the two methods (% agreement = 99.9%). The SWV results were also within +/- 1.0 standard deviation of the target values of L-Phe within the DBS samples, thus indicating the high accuracy of the sensor. These results warrant large scale clinical trials to complete the optimisation/validation of the new immunosensor.

## Materials and methods

### Chemicals and reagents

L-Phenylalanine (L-Phe, 99%, CAS: 63–91-2) and corresponding recombinant monoclonal antibody (JE64-47) were purchased from Thermo Fisher (Australia). D-phenylalanine (98%) was purchased from Sigma Aldrich (USA). Potassium hexacyanoferrate (II) trihydrate (K_4_[Fe(CN)_6_] ·3H_2_O, 98.5%), phosphate buffer saline tablets (PBS), L-tryptophan (98.0%), L-tyrosine (99%0.0), L-alanine (99.0%), 3-mercaptopropionic acid (99.0%) and gold (III) chloride trihydrate (99.9%) were purchased from Sigma (USA). Quality control human DBS on filter paper cards were obtained from the 2023 ERNDIM External Quality Assurance Scheme (UK) [18]. The concentration of L-Phe in the DBS samples are certified in the ERNDIM annual report 2023 [19].

Screen-printed gold electrodes (SPE, 250BT) were purchased from Metrohm (Australia).

### Instrumentation

CA, cyclic voltammetry (CV), and SWV measurements were carried out using the handheld PalmSens 3 potentiostat and a 4 mm banana connector (PalmSens, Netherlands) to connect the SPE to the potentiostat. The software PSTrace 5.11 (PalmSens, The Netherlands), was used to control the electrochemical measurements. The electrochemical measurements were reported without data processing or background subtraction procedure.

For POC demonstration, a Sensit Smart potentiostat (PalmSens, The Netherlands) was plugged into the USB-C port of a smart phone and the new immunosensor was installed into the potentiostat sensing port. The potentiostat was operated using the PSTouch v2.9 software (PalmSens, The Netherlands).

Scanning Electron Microscope equipped with an Energy Dispersive detector (SEM-EDS, Zeiss Sigma FESEM, Germany) was used for the characterisation of the gold nanostructures on the SPE. Atomic force microscope (Bruker Dimension Icon, USA) was used for imaging the surface of the immunosensor after gold deposition, antibody attachment and L-Phe binding.

Raman measurements were carried out within the wavelength range 400–1800 cm^− 1^ using a handheld Raman spectrometer (Ocean Optics, USA) at a spectral resolution of 12 cm^− 1^. The background noise and fluorescence interference were automatically corrected using OceanView Spectroscopy v1.5.07 software.

For light-driven surface functionalisation, an LED light source was used to generate a 280 nm beam. The light source was mounted on a metal-core printed circuit board (MCPCB-mounted LED light, model M280D4, Thorlabs, USA) and controlled by a T-Cube LED Driver (Model LEDD1B, Thorlabs, USA). The LED driver was connected to the light source using a connection cable (CAB-LEDD1, Thorlabs, USA).

### Fabrication and characterisation of gold-nanostructures sensor

An SPE was connected to the potentiostat using a banana connector. The working electrode of the SPE was cleaned and activated by CV (10 cycles) in 0.1M H_2_SO_4_ solution using a scan rate of 0.1 V/s, (5 scans per measurement), potential = 0.0024 V, a current = 1 mA, equilibration time = 10 s and a potential sweep from 0.6 V to −0.6 V. Potassium hexacyanoferrate (0.05 M in 1x PBS, pH 7.4) was used as an electrolyte.

After cleaning, the SPE was immersed into a vial containing 3 ml of 4 mM HAuCl_4_ (in 0.1 M perchloric acid) as an electrolyte. Gold nanostructures were deposited on the SPE using CA that was carried out using the following parameters: DC voltage = −0.08 V E dc, time interval = 0.1 s, and equilibration time = 2 s. The gold deposition was carried out for 100 s, 300 s and 600 s respectively. After deposition, the SPE was thoroughly rinsed with deionized water then dried under nitrogen gas. Characterisation before and after 600 s gold deposition was carried out by SEM and SEM-EDS.

To determine the conductivity of the sensor after the deposition of gold nanostructures, CV measurements were carried out using a scan rate of 0.1 V/s, (5 scans per measurement), potential = 0.0024 V, potential sweep from 0.6 V to −0.6 V, current = 1 mA, and equilibration time = 10 s. Potassium hexacyanoferrate (0.05 M in 1x PBS, pH 7.4) was used as an electrolyte for the electrochemical measurements.

### Preparation of standard solutions

10 mM stock solutions of L-tryptophan, L-tyrosine and L-alanine (100 mL each) were prepared by weighing 0.204 g L-tryptophan, 0.181 g L-tyrosine and 0.089 g L-alanine in separate measuring flasks and dissolving the powder in 100 mL of 1x PBS (pH 7.4). L-Phe stock solution (10mM) was prepared by dissolving 0.165 g of the amino acid into 100 mL of 1x PBS (pH 7.4). A series of L-Phe standard solutions (2000 µM, 1200 µM, 600 µM, 300 µM, 100 µM, 50 µM, 5 µM, 0.5 µM and 0.1 µM) were prepared by serial dilution from the stock solution using 1x PBS (pH7.4). JE64-47 antibody dilute solution (1 × 10^− 7^ M) was prepared by accurately diluting 100 µL of the supplied antibody stock solution with 6.66 mL of 1x PBS (pH 7.4).

### Development of a L-Phe electrochemical immunosensor

20 µL of the antibody solution (1 × 10^− 7^ M) were loaded onto the gold-nanostructured sensor and exposed to a LED light @280 nm. The light source was operated at 75% intensity and 0.35 A for 30 min to reduce the disulfide bonds within the antibody molecules. After antibody attachment, the electrode was rinsed three times with 1x PBS (pH 7.4) and gently dried using nitrogen gas. The antibody attachment onto the SPE was confirmed by Raman spectroscopy using the handheld Raman spectrometer. The sample excitation was carried out using a 785 nm laser beam that delivers 5 mW laser power at the sample. Five accumulations (time per accumulation = 100 ms) and 0.5 s total acquisition time were used to acquire a Raman spectrum. The Raman measurements were carried out using the raster orbital scanning mode (ROS) to acquire an average Raman spectrum of the probed sample [20].

To prevent nonspecific adsorption of interfering molecules, the remaining bare sites on the gold nanostructures were blocked using 20 µL of 1 × 10^− 8^ M mercaptopropionic acid (MPA). The acid was loaded onto the electrode surface and allowed to stand for 40 min. The SPE was then rinsed five times with 1xPBS (pH 7.4). CV measurements were carried out to determine the change in the SPE current and potential after antibody immobilization and surface blocking. CV measurements were caried using a scan rate of 0.1 V/s, (5 scans per measurement) at a potential of 0.0024 V, 1 mA current, 10 s equilibration time and a potential sweep was from 0.6 V to −0.6 V. Potassium hexacyanoferrate (0.05 M in 1x PBS, pH 7.4) was used as an electrolyte.

### Control tests and selectivity of the new L-Phe electrochemical immunosensor

To determine the selectivity of the functionalised immunosensor, negative and positive control samples were prepared. The negative control sample that contains 300 µM of each of L-tryptophan, L-tyrosine, and L-alanine. This was prepared by mixing equal volumes of their respective 10 mM stock solutions and diluting to 1 mL with 1x PBS (pH 7.4). The positive control sample was prepared by mixing of 30 µL of L-tryptophan, L-tyrosine, and L-alanine (300 µM each) with 15µL of L-phenylalanine (10 mM) and completing the volume to 1 mL using 1x PBS (pH 7.4). The final concentration of L-phenylalanine in the positive control sample was 150 µM. 20 µL aliquots of the negative and positive control samples were loaded onto two immunosensors and allowed to stand for 15 min. The immunosensors were then rinsed with 1x PBS (pH 7.4) to remove unbound amino acids. CV measurements were carried out as described earlier and the magnitude of the oxidation and reduction currents were recorded.

To determine the selectivity of the electrochemical immunosensor towards L-Phe over D-Phe, 300 µM of D-Phe was prepared by dissolving 5.05 mg of the amino acid in 100 mL of deionised water. A 20 µL aliquot of the D-Phe was loaded onto two immunosensors and allowed to stand for 15 min. The immunosensors were then rinsed with 1x PBS (pH 7.4) to remove unbound amino acids. CV measurements were carried out as described earlier and the magnitude of the oxidation and reduction currents were recorded.

### Quantification of L-Phe using the new electrochemical immunosensor and SWV

20 µL aliquots of L-Phe solutions within the concentration range 2000 µM to 0.1 µM were loaded on 3 mm filter paper discs. The discs were placed in the wells of a 96 well plate separately and allowed to air-dry for 24 h. 100 µL aliquots of 1x PBS (pH 7.4) were loaded into each well and allowed to stand for 1 h at room temperature, with gentle agitation every 5 min to extract the L-Phe from the paper discs. 20 µL aliquots of the L-Phe extract (in 1x PBS, pH = 7.4) were loaded onto individual immunosensors and allowed to stand for 15 min. The immunosensors were then washed thoroughly with 1x PBS (pH 7.4), dried under a nitrogen stream, and analysed by SWV using the following parameters: current = 1 mA, potential sweep from − 0.2 to 0.5 V, step = 0.0024 V, amplitude = 0.09 V and frequency = 25.0 Hz frequency. The SWV measurements were carried out using potassium hexacyanoferrate solution (0.05 M in 1x PBS, pH 7.4) as the electrolyte.

### Determination of L-Phe in DBS by the electrochemical immunosensor

The new immunosensor was used to determine the concentration L-Phe in eight DBS samples from ERNDIM. The measurements were carried out with approval from the ethics committee at Queensland University of Technology (approval number 8454). 3 mm discs were cut off DBS cards and loaded into the wells of a 96 well plate. 100 µL aliquots of 1x PBS (pH 7.4) were added to the wells with gentle agitation to extract the amino acids from the dry blood spot. 20 µL aliquots of the L-Phe extract (in 1x PBS, pH = 7.4) were loaded onto individual immunosensors and allowed to stand for 15 min. The immunosensors were then washed thoroughly with 1x PBS (pH 7.4), dried under a nitrogen stream, and analysed by SWV as described previously.

## Results and discussion

### Fabrication of gold nanostructured sensor

Electrochemical sensors have been demonstrated for the screening of various biomolecules [21, 22]. To develop an efficient electrochemical immunosensor for disease biomarkers, the sensitivity and selectivity of the sensor must be maximised. This can be achieved by modifying the sensor surface to increase its electroactive surface area and functionalise it with target-specific recognition molecules that selectively bind the target analyte from complex sample matrices. Prior to functionalization, the electrode surface requires pretreatment to remove any contaminants. The cleaning process was carried out using cyclic voltammetry (10 cycles) and sulfuric acid (0.1 M) as an after eight CV cycles, thus indicating the removal of surface contaminants (Fig S1). This was confirmed by SERS measurements of the electrode surface before and after CV cleaning (Fig S1). As shown by the figure, no Raman signals were observed on the surface of the SPE after the CV cleaning process (8 cycles).

**Fig. 1:**
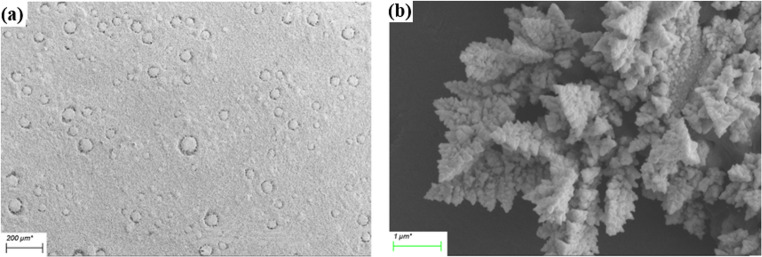
SEM of (**a**) SPE electrode and (**b**) gold nanostructures on SPE after electrodeposition for 600 s by CA

In this work we deposited gold nanostructures onto an SPE by CA to increase its electroactive surface area (Fig S3 (a)). The gold nanostructures deposition was carried out for 100 s, 300 s, and 600 s to determine the optimum deposition time that can provide a highly electroactive sensor. As shown by Figure S1 (b), a notable increase in the sensor conductivity (as indicated by the increase in the sensor electric current and the shift in oxidation/reduction potentials from + 0.31 V to + 0.17 V, and from − 0.03 V to + 0.07 V) was observed after the deposition of gold nanostructures for 600 s. The SEM images of the sensor surface after gold nanostructures deposition for 600 s showed the formation of forests of fern-like gold nanostructures (Fig. 1a, b). The electrodeposition of fern-like gold nanostructures by chronoamperometry is attributed to a diffusion-limited nucleation of gold seeds that grow into fern-like gold nanostructures on the SPE surface [23]. Unlike other electrodeposition methods that have been demonstrated in the literature, our method doesn’t utilize a shaping agent (e.g. PEG) to influence the formation of the fern-like gold nanostructures [24]. Instead, we utilize electrochemical force to drive kinetically controlled growth of the deposited gold seeds into fern-like nanostructures [25]. Additionally, the EDS measurements confirmed the high increase in the gold metal concentration on the electrode surface after the deposition process (Au % = 77.2%, Figure S4). Therefore, the increase in the sensor conductivity can be attributed to the increase in its electroactive surface area due to the deposition of the gold nanostructures.

### Development of L-Phe electrochemical immunosensor

The fern-like gold nanostructures were functionalised with target-specific antibody recognition molecules without the use of chemical reagents that can compromise the antigen recognition property of the antibody and cause environmental pollution. LED light @280nm was utilised to break the disulfide bonds within the hinge region of the antibody [26]. The energy of the 280 nm photons is absorbed by the cysteine residues near the disulfide bonds, thus leading to their reduction and the formation of free sulfhydryl (SH) groups. Au-S bonds form between the reduced antibody fragments and the gold nanostructures on the sensor. The attached antibody fragments assume a unified upward trajectory on the electrode surface that facilities their binding of the L-Phe molecules [27]. The attachment of the antibody molecules to the gold nanostructures of the SPE was confirmed by SERS measurements where characteristic Raman bands of antibody structure were detected on the sensor surface after the antibody attachment process (Figure S5 and Table S1) [28].

MPA molecules were used to backfill the remaining bare gold nanostructures on the immunosensor surface. The MPA molecules form A-u-S bonds with the gold nanostructures. The negatively charged carboxyl groups of MPA exert repulsion forces against interfering molecules and hinder their nonspecific adsorption to the sensor surface, thus maximizing its selectivity [29, 30].

**Fig. 2:**
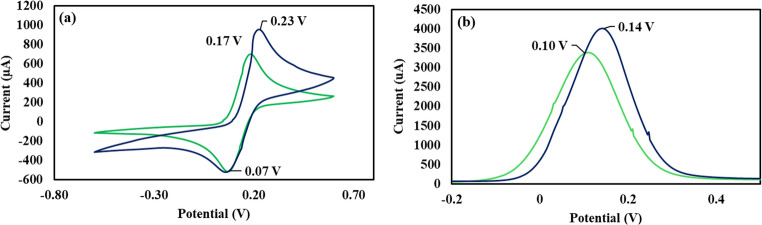
(**a**) Cyclic voltammetry and (**b**) square wave voltammetry of the gold nanostructured SPE before (green) and after antibody functionalisation/backfilling (blue)

The electrochemical immunosensor was screened by CV and SWV after antibody attachment and backfilling (Figure S6, Fig. 2a, b). As shown by the figure, the electric current and oxidation potential of the immunosensor shifted to 0.23 V in the CV measurement and to 0.14 V in the SWV measurement. These changes can be attributed to the formation of a mixed MPA/antibody negatively charged monolayer on the gold nanostructures that facilitates the electron flow towards the sensor conductive surface [31–33].

### Surface analysis of the L-Phe immunosensor by AFM

**Fig. 3:**
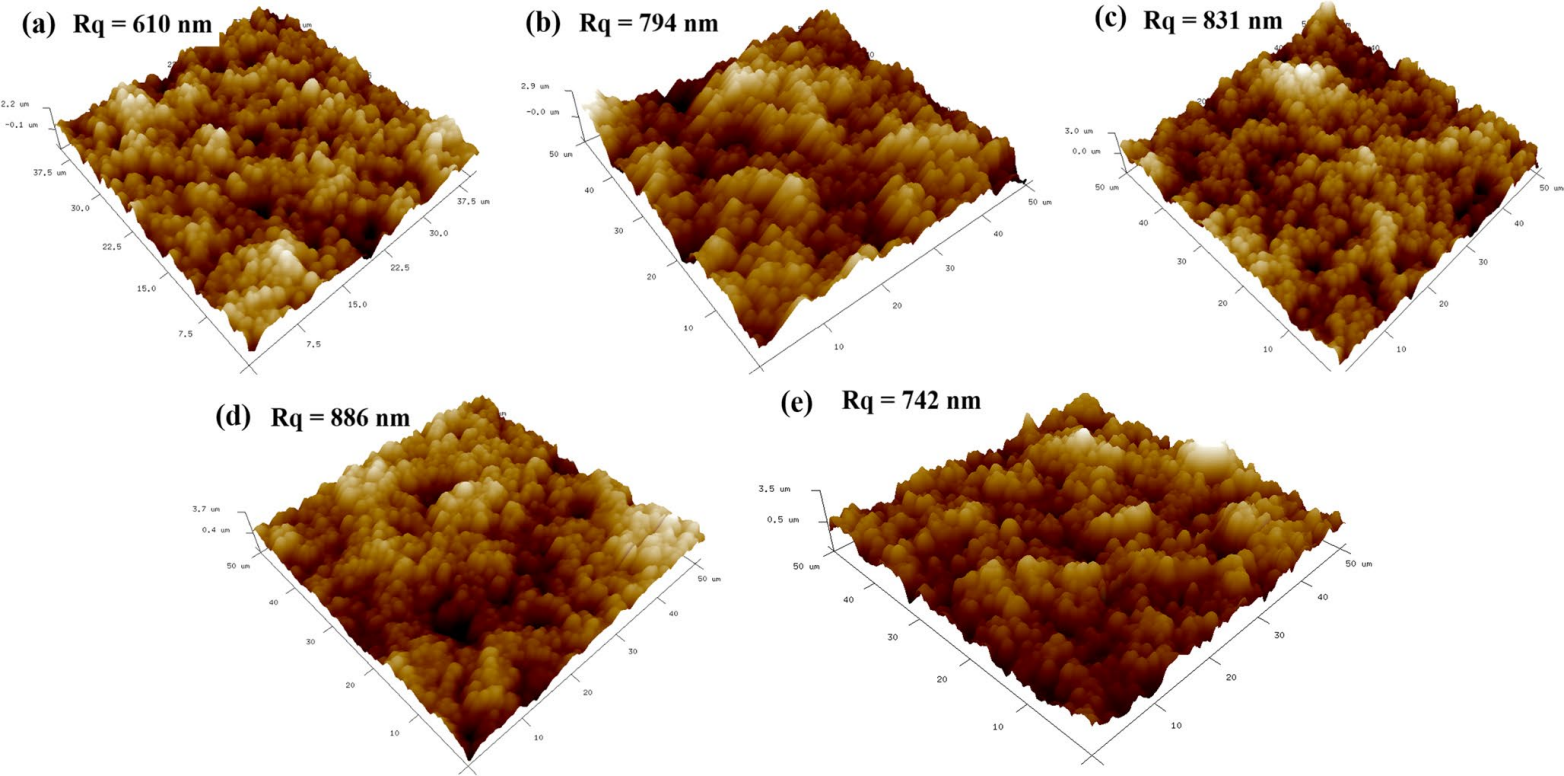
AFM images of (**a**) bare SPE (**b**) gold-nanostructured sensor (**c**) gold-nanostructured sensor after antibody attachment (**d**) and after backfilling with MPA and (**e**) after binding with L-Phe

The roughness of the immunosensor surface after antibody attachment, backfilling and L-Phe binding were investigated by AFM (Fig. 3). As indicated by the figure, the root mean square roughness (Rq) of the SPE increased from 610 nm to 794 nm after the deposition of the gold nanostructures, which can be attributed to the irregular shape of the nanostructures. After antibody attachment, the Rq of the SPE increased to 831 nm, which can be attributed to upward trajectory that the attached antibody molecules assume on the sensor surface [33, 34]. After backfilling, the Rq increased to 886 nm. This can be attributed to the electrostatic repulsion between the MPA and antibody molecules on the sensor surface. The Rq of the immunosensor decreased to 742 nm after binding the L-Phe which can be attributed to the formation of antibody-ligand complex that changes the antibody orientation to a lie -down trajectory [33, 35]. The corresponding SEM images for the antibody attachment, backfilling, and L-Phe binding steps are shown in Fig. S7. Elemental analysis shows a decrease in the gold signal and an increase in carbon content, consistent with the addition of organic molecules onto the gold nanostructure surface.

### Selectivity of the new immunosensor towards L-Phe

**Fig. 4:**
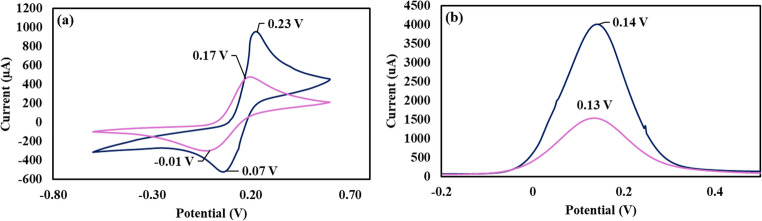
(**a**) CV and (**b**) SWV of the antibody-functionalised/backfilled immunosensor before (blue) and after binding L-Phe (purple)

The binding between L-Phe and the new immunosensor was determined by CV and SWV (Fig. 4a, b). As indicated by the CV measurement (Fig. 4a), when the immunosensor binds L-Phe, its electric current reduces significantly, and the oxidation and reduction potentials shift to 0.17 V and 0.01 V respectively. Similarly, the electric current reduces significantly in the SWV measurement and potential shifts to 0.12 V (Fig. 4b). The reduction in the sensor current can be attributed to the formation of the antibody-antigen complex which takes a lie down position that shield the immunosensor conductive surface and reduces the electron transfer [36].

**Fig. 5:**
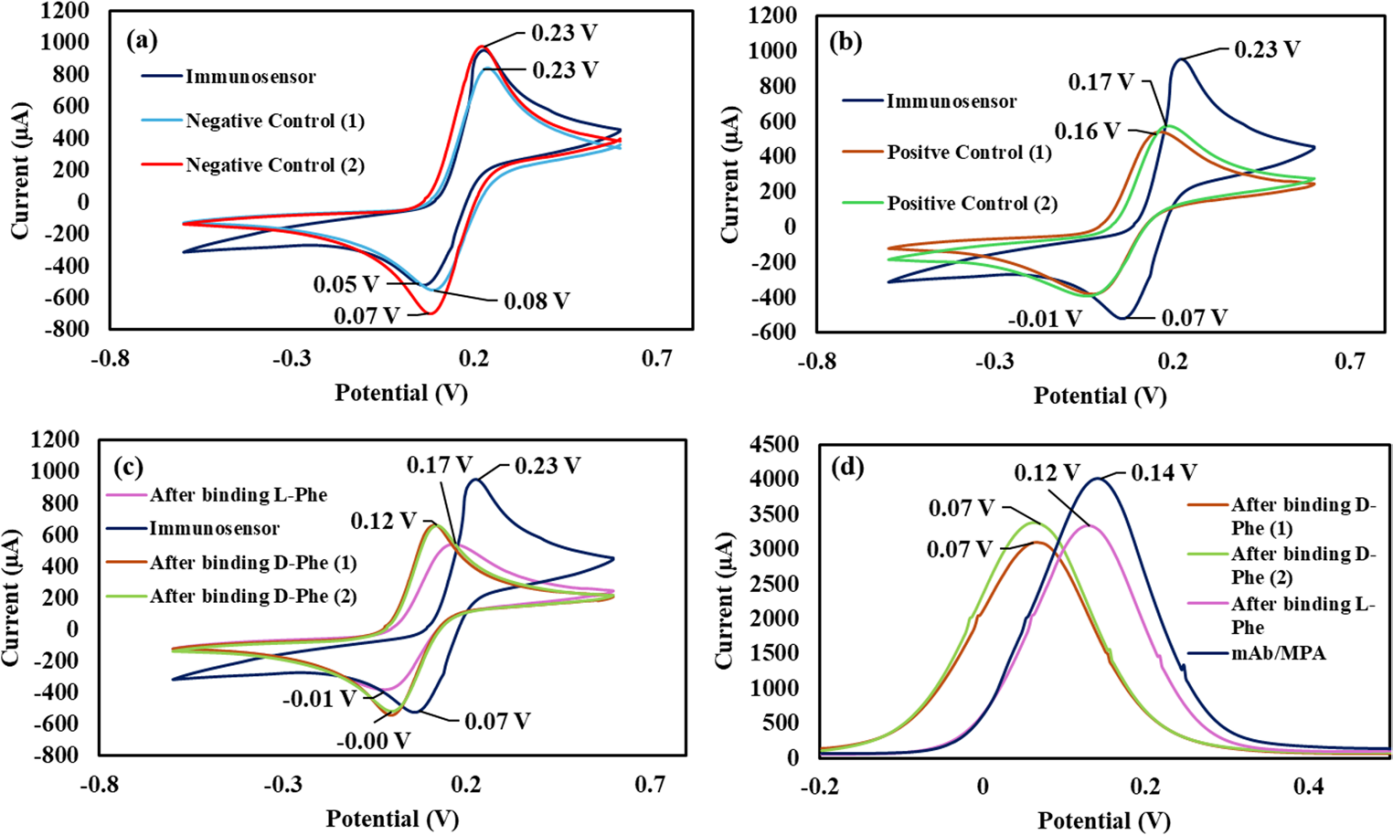
CV of the immunosensor after binding L-Phe from (**a**) negative control and (**b**) positive control samples, (**c**) CV and (**d**) SWV of the immunosensor before and after binding D-Phe

To confirm the selectivity of the immunosensor towards L-Phe, negative and positive control samples were screened by CV. As shown by Fig. 5a, the CV of the immunosensor did not change upon interaction with the negative control sample. In contrast, the CV of the immunosensor the electric current and oxidation and reduction potentials changed similar the changes that occurred upon binding between the immunosensor and L-Phe standard solution (Fig. 5b). These results demonstrate the high selectivity of the immunosensor towards L-Phe in the presence of structurally related amino acids such as L-tryptophan, L-tyrosine and L-alanine.

To further confirm the high selectivity of the immunosensor towards the L-Phe isoform, it was utilised to bind D-Phe from standard aqueous solution. After binding, the immunosensor was screened by CV and SWV (Fig. 5c, d). As indicated by the figure, the binding of D-Phe caused the sensor potential to shift to 0.12 V in the CV measurement and to 0.07 V in the SWV measurement. Therefore, there was a significant difference in the sensor response towards L-Phe (Fig. 4a, b) and D-Phe (Fig. 5c, d), and it can be used for the determination of L-Phe without interference from D-Phe.

### Electrochemical quantification of L-Phe by the new immunosensor

**Fig. 6:**
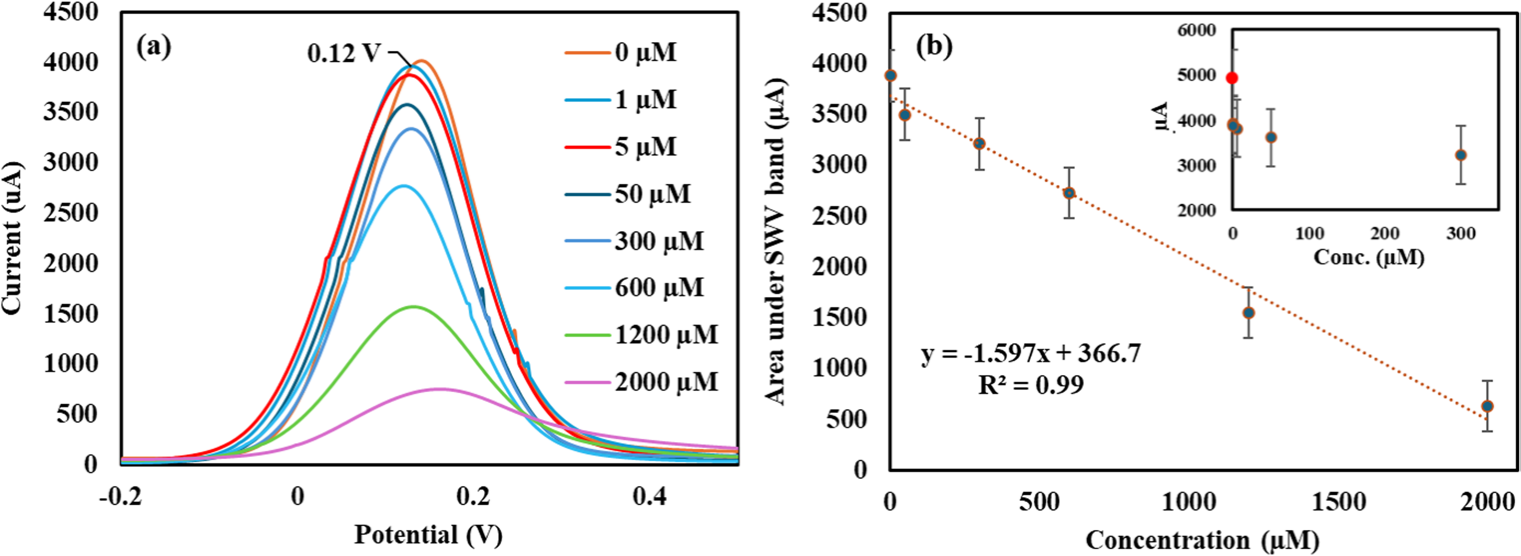
(**a**) SWV Voltammograms of different concentrations of L-Phe and (**b**) the corresponding calibration plot of L-Phe by SWV. The inset depicts the SWV of the immunosensor in the L-Phe concentration range 0.1 µM to 300 µM

The new immunosensor was used to quantify L-Phe within the clinical range 120 µM-1200 µM by SWV [37]. The SWV method was selected due to its rapid screening time (< 10 s), and high sensitivity. As shown by Figs. 6(a, b) the magnitude of the current in the SWV measurements changed proportionally with the concentration of L-Phe within the range 0.1µM and 2000 µM (inset of Fig. 6b). A linear correlation was observed between the concentration of L-Phe and the area under the SWV band within the range 1 µM to 2000 µM. This relationship was found to follow the linear regression equation y = −1.597x + 366.76, and the correlation coefficient (R^2^) was 0.99. The lowest limit of quantification (LOQ) by SWV was determined experimentally to be 1µM. The lowest limit of detection of L-Phe by SWV was calculated using the equation LOD = 3/Sσ​ (σ = standard deviation of the blank, *m* = slope of the calibration plot) and found to be 0.3 µM.

The shelf life of the L-Phe immunosensor was determined by monitoring the change in its current over 14 days using SWV. As shown by Figure S8, the current of the immunosensor decreased by 5.96% over a 14-day period, thus indicating good stability of the immunosensor. This can be attributed due to the inert nature of the immunosensor gold nanostructures and the covalent bonding of the antibody and MPA molecules to its surface. In addition, three independent immunosensors were prepared and used to screen L-Phe standard solution (600 μm) by SWV (*n* = 9). As shown by Figure S9 (a, b), the RSD within the SWV measurements was 1.69, thus indicating the high reproducibility of the new immunosensor fabrication process.

### Determination of L-Phe in DBS samples by the new immunosensor

Th new immunosensor was used to determine the concentration of L-Phe in eight quality control DBS samples from ERNDIM by SWV (Figure S10). The SWV measurements were compared to the certified concentrations of L-Phe [19]. As indicated by Table 1, there was a good agreement of between the certified L-Phe concentrations and those determined by SWV (% agreement = 90.9% − 111.2%), with a mean relative standard deviation (RSD) of 1.4% (*n* = 2). Also, the immunosensor showed excellent linearity over the clinical concentration range of 75–1975 µM (regression equation: y = 0.9212x + 36.342, R^2^ = 0.99) (Fig. S11).

**Table 1:** Concentrations of each DBS sample as measured by SWV

DBS No.	Conc. of L-Phe by ERNDIM (µM)	Area under SWV curve (µA)	Conc. of L-Phe as measured by SWV (µM)	RSD value (%) (*n* = 2)	% agreement
1	1975.9	648	1890.4	4.8	95.6
3	975.8	2166	939.7	0.9	96.3
5	477.0	2926	464.6	1.9	97.1
7	75.1	3549	74.3	0.4	98.9
9	975.8	2119	968	0.7	99.2
11	477.0	2823	530.4	0.5	111.2
13	1975.9	803	1795.7	1.6	90.9
15	75.1	3535	82.82	0.2	110.3
	Average			1.4	99.9

The accuracy of the SWV method was confirmed by comparing the results from Table 1 to the mean values from all participants in the ERNDIM quality assurance program (Table S1). The z-scores for each sample were calculated using the formula (SWV results - mean)/SD. All z-scores fell within the ± 1.0 range, while scores within ± 2.0 were deemed acceptable. The precision was determined using the paired values in the samples (i.e. samples 7 and 15, samples 5 and 11, samples 3 and 9, and samples 1 and 13). The CV% were determined to be 7.67%, 7.50%, 2.10% and 3.63% at 78.56, 503.70, 953.85 and 1843.05 µmol/L phenylalanine accordingly (Table S3).

**Fig. 7:**
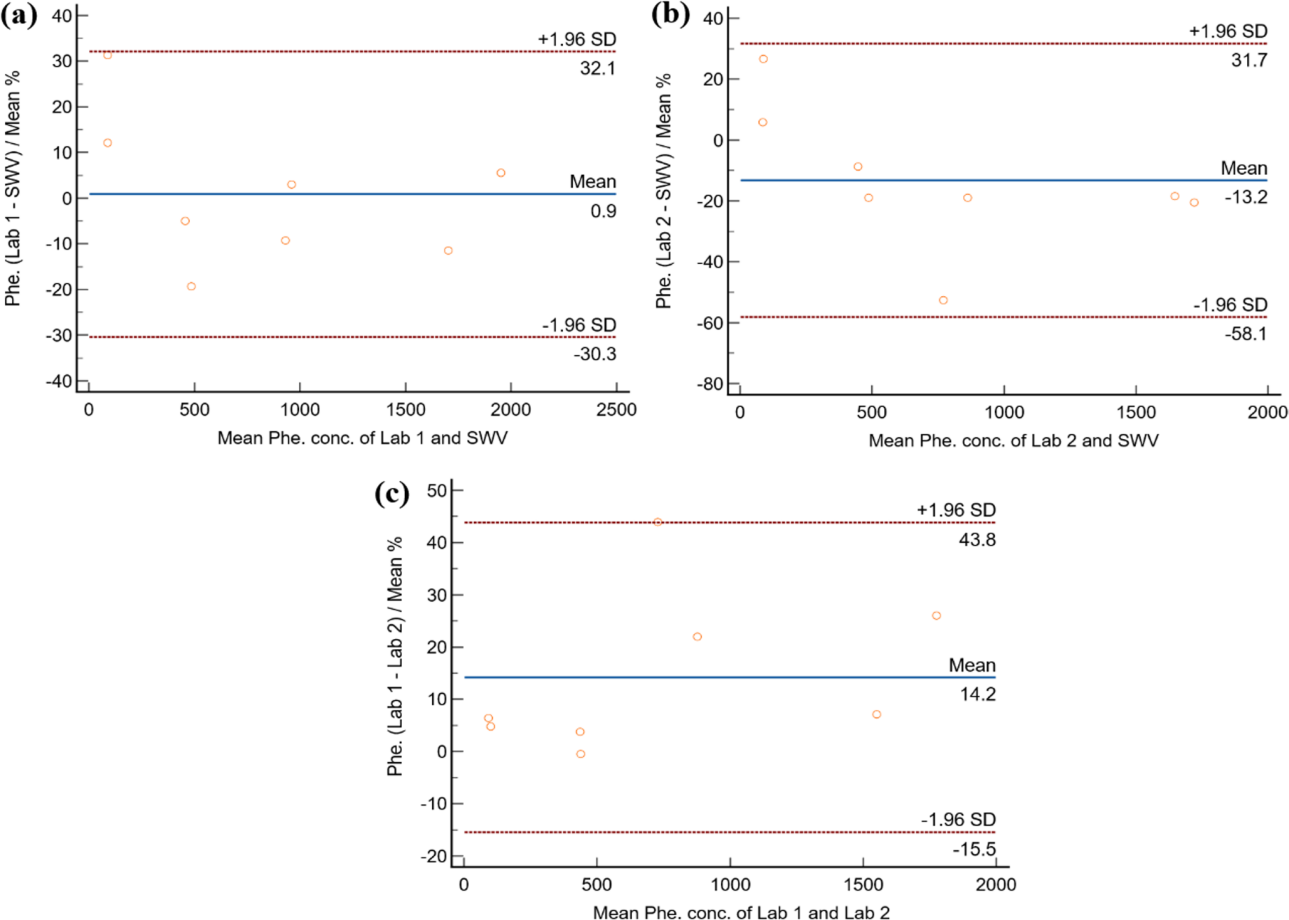
Bland-Altman plot analysis for L-Phe in human blood dry spot determination by (**a**) SWV and Lab 1 (**b**) SWV and Lab 2 and (**c**) Lab 1 and Lab 2

The concordance of the SWV measurements was assessed using Bland-Altman plot analysis (Fig. 7a-c). The Bland-Altman method was chosen as a statistical technique to differentiate between the two quantitative measurement methods [38]. Statistically, there was no significant difference between the two methods, with a mean percentage difference of zero (p-value = 0.8417). In contrast, a low p-value (e.g., < 0.05) would indicate that the mean percentage difference is statistically significant. The mean percentage difference was found to be −0.53%, with a 95% confidence interval of −6.61 to 5.54 across the tested concentrations, ranging from approximately 75 to 2000 µmol/L. In clinical practice, mass spectrometry is one of the most used methods for quantifying phenylalanine. We compared the performance of the SWV method with two mass spectrometry assays from two independent pathology labs (laboratories 1 and 2, Fig. 7). The percentage differences were determined to be 0.88% (95% CI: −12.44 to 14.21%) for laboratory 1 and − 13.22% (95% CI: −32.37 to 5.94%) for laboratory 2.

**Fig. 8:**
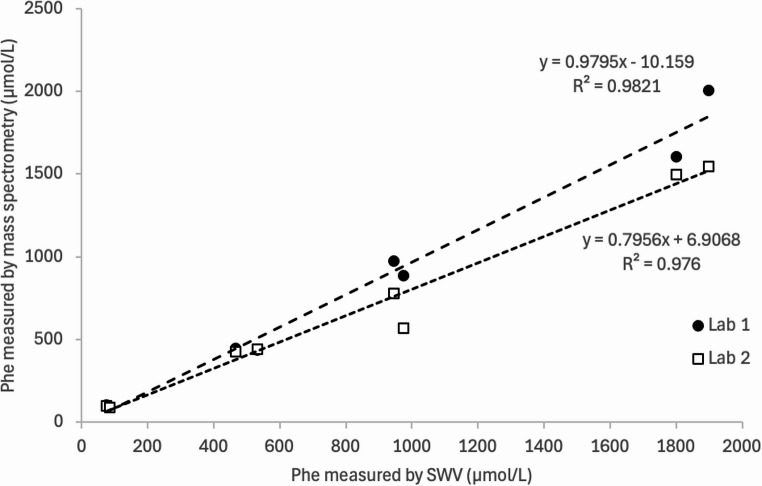
Linear regression model of L-Phe measured by SWV and mass spectrometry from the 2 laboratories

Linear regression analysis was used to compare the SWV response with the Lab 1 measurements, as shown in Fig. 8. The resulting regression equation *y* = 0.9795*x*−10.159, with a coefficient determination (R^2^) of 0.9821. For the data from SWV and Lab 2, the regression equation is *y* = 0.7956*x* + 6.9068, with a coefficient of determination (R^2^) of 0.976 where *y* represents data from SWV and *x* represent data from mass spectrometry.

To demonstrate the potential new immunosensor for POC application, it was used to qualitatively detect the presence of L-Phe in a DBS sample by SWV, and an ultra-compact potentiostat and a smartphone (Figure S12). As shown by the figure, the sensor current reduced significantly and shifted to 0.12 V after its interaction with the DBS sample. These changes were like those observed in Fig. 4b and confirm the successful binding of the amino acid.

## Conclusion

This study presents a proof of concept on a novel, highly sensitive, and target-specific L-Phe immunosensor for L-Phe in PKU patients. The new electrochemical immunosensor was synthesized by modifying an SPE with fern-like gold nanostructures, to increase its electroactive surface area, and decorating the nanostructures with target-specific antibody recognition molecules using a novel light driven surface functionalisation process that has low carbon footprint on the environment. The new sensor directly detects L-Phe without the need for electroactive reporter can differentiate between L-Phe, D-Phe and other endogenous biomolecules, thus maximising its selectivity and sensitivity for L-Phe quantification by SWV. The new immunosensor showed a good linearity when used for L-Phe quantification by SWV within the concentration range between 1 µM to 2000 µM (LOD = 0.3 µM, LOQ = 1 µM, R^2^ = 0.99). Therefore, it was utilised for the screening of L-Phe in human dry blood spots, and the results showed 99.9% (+/- 1.0) agreement with the target values. The SWV measurements showed no statistical difference from Mass spectroscopy screening that were carried out by two independent laboratories. The new sensor was integrated with an ultra-compact potentiostat in a Lab-on-Phone assembly and used to screen DBS samples, thus indicting the sensor potential for POC and at-home screening for PKU patients. This assembly has the potential for real time transmission of results to the medical doctor for timely medical intervention for patients in urban and remote locations, where access to diagnostic services may be limited. The sensor high sensitivity across the clinically relevant range (120–1200 μm) indicates its potential for screening L-Phe in newborns and PKU patients. Therefore, these results warrant a large-scale clinical trial to further validate the new immunosensor for POC and at home screening and for advance it towards a wearable monitoring device that provides timely insights into the metabolic flux of phenylalanine in PKU patients.

## Supplementary Information

Below is the link to the electronic supplementary material.

Supplementary Material 1 (DOCX 1.77 MB)

## Data Availability

All data supporting the findings of this study are available within the paper and its Supplementary Information.
