# Supplementary material for: Ultra-sensitive nanostructured electrochemical immunosensor for selective monitoring of L-phenylalanine in phenylketonuria patients
Source: Mikrochim Acta. 2026 Jan 23;193(2):106. doi: 10.1007/s00604-026-07836-8 (PMC12830426; doi:10.1007/s00604-026-07836-8)
Supplement: Supplementary file 1 — Supplementary Material 1 (DOCX 1.77 MB) [file 604_2026_7836_MOESM1_ESM.docx]

Ultra-sensitive nanostructured electrochemical immunosensor for selective monitoring of L-phenylalanine in Phenylketonuria Patients

Rebecca L. Houston^1^, Eric CY. Law^2,3^, & Emad L. Izake^1,4^

1. School of Chemistry and Physics, Queensland University of Technology, Brisbane, Queensland, Australia

2. The Children’s Hospital at Westmead, Sydney, New South Wales, 2145, Australia

3. The University of Sydney, Camperdown, New South Wales, 2050, Australia

4. Centre for Materials Science, Queensland University of Technology (QUT), 2 George Street, Brisbane, QLD, 4000, Australia

- Corresponding author email address: [rebeccca.houston@hdr.qut.edu.au](mailto:rebeccca.houston@hdr.qut.edu.au)


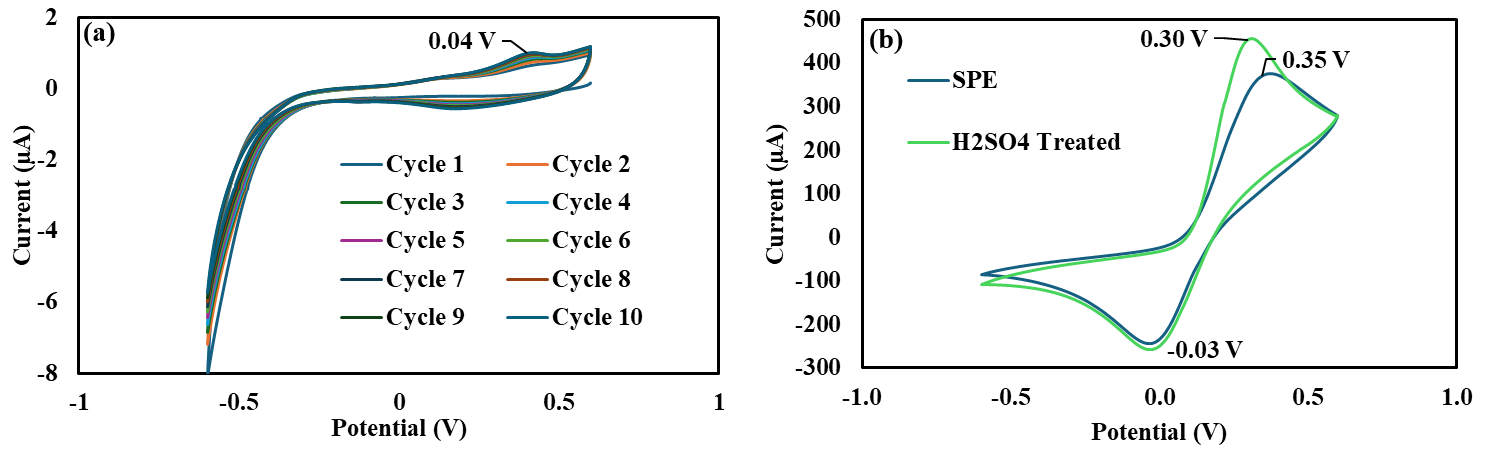


Fig.S1(a) CV of cleaning the SPE in 0.1M H_2_SO_4_ and (b) CV of the SPE before (blue) and after cleaning with 0.1M H_2_SO_4_ (green).


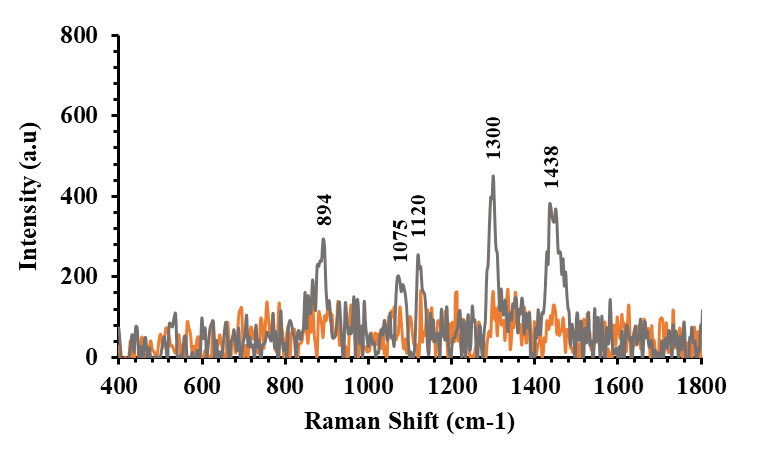


Fig.S2 Surface analysis of the SPE before (grey) and after (orange) cleaning with 0.1M H_2_SO_4_.


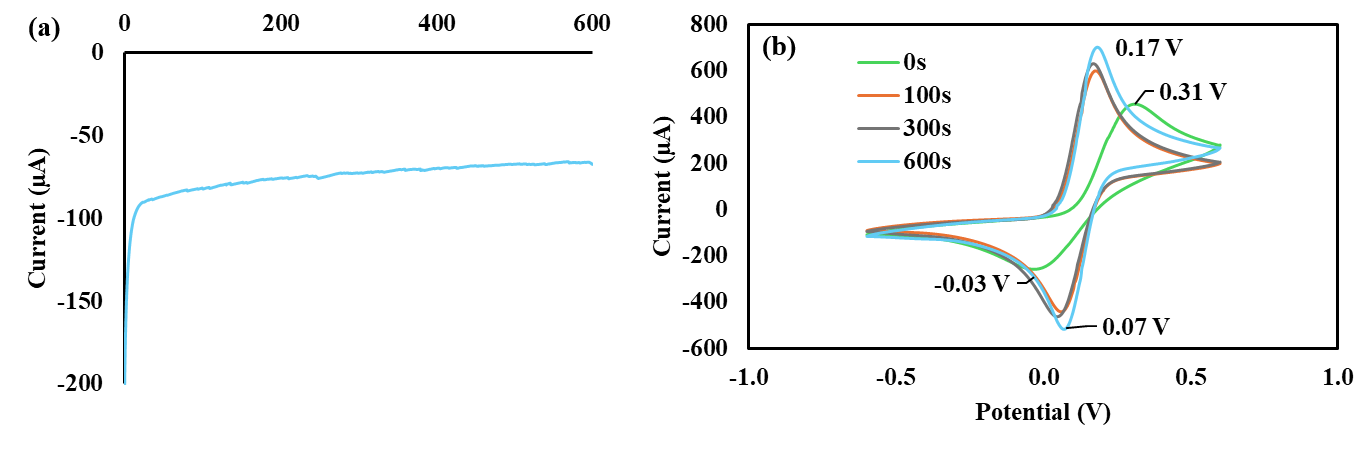


Fig.S3. (a) CA for 600 s gold deposition and (b) CV of the SPE after gold deposition by CA for 0 s, 100 s, 300 s and 600 s.


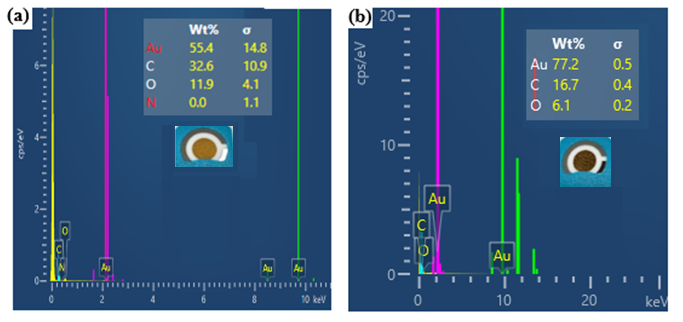


Fig.S4. EDS elemental analysis of the SPE before (a) and (b) after gold nanostructures deposition for 600 s.


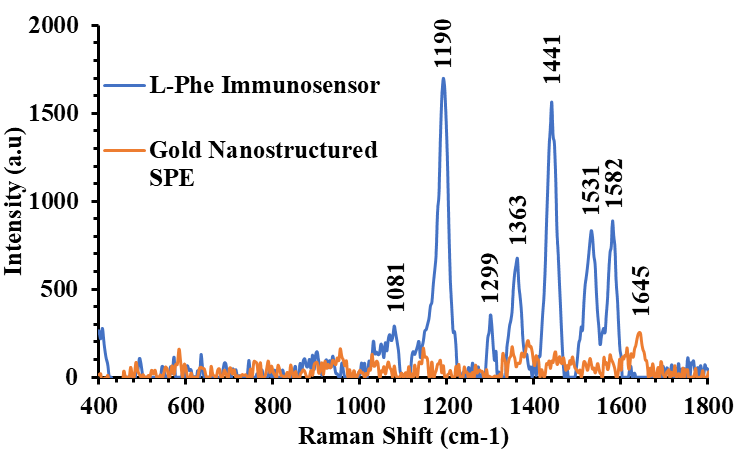


Fig.S5. SERS measurements of the L-Phe immunosensor after light-driven attachment of the antibody molecules.

Table S1, Band assignment of the SERS spectra of reduced monoclonal antibody.

| **Raman Shift (cm-1)** | **Band Assignment** | **References** |
| --- | --- | --- |
| 1081 | Phenyl -CH plane bending, C-N vibrations | [1, 2] |
| 1190 | C-H bending aromatic side chain | [3] |
| 1299 | Amide III C-N stretch N-H bend | [4] |
| 1363 | CO stretching, COOH stretch | [5] |
| 1441 | C-H2 bending, cysteine residues | [4, 6] |
| 1531 | Amide II N-H bend, NH_3_^+^ stretching | [7] |
| 1582 | C=C stretch of aromatic ring | [8] |


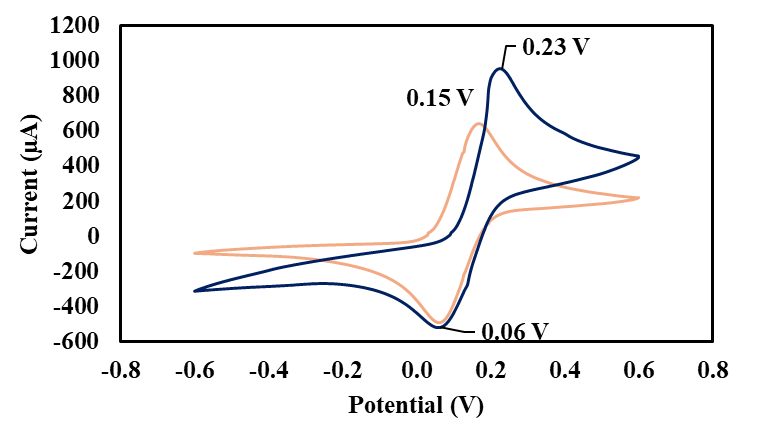


Fig S6. Cyclic voltammetry of the immunosensor after antibody attachment (orange) and after backfilling with MPA (navy).


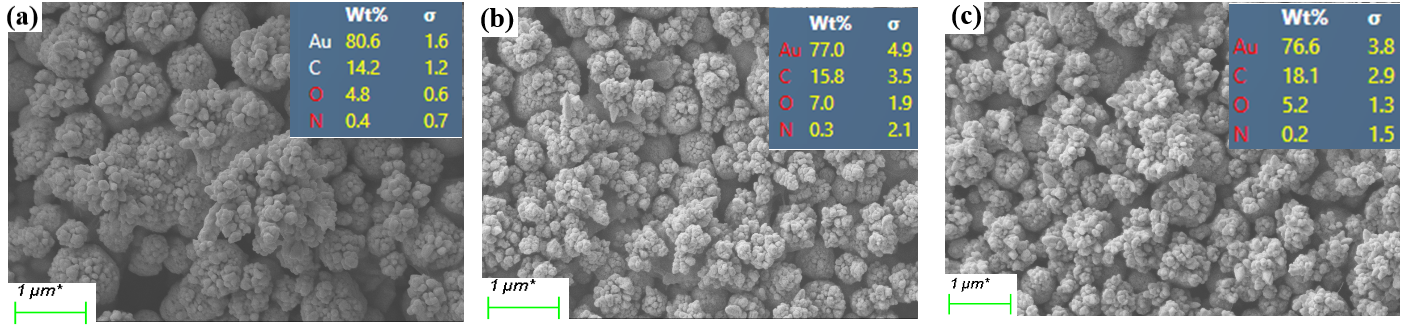


Fig S7. SEM-EDS images (a) after antibody attachment (b) MPA backfilling and (c) L-Phe binding.


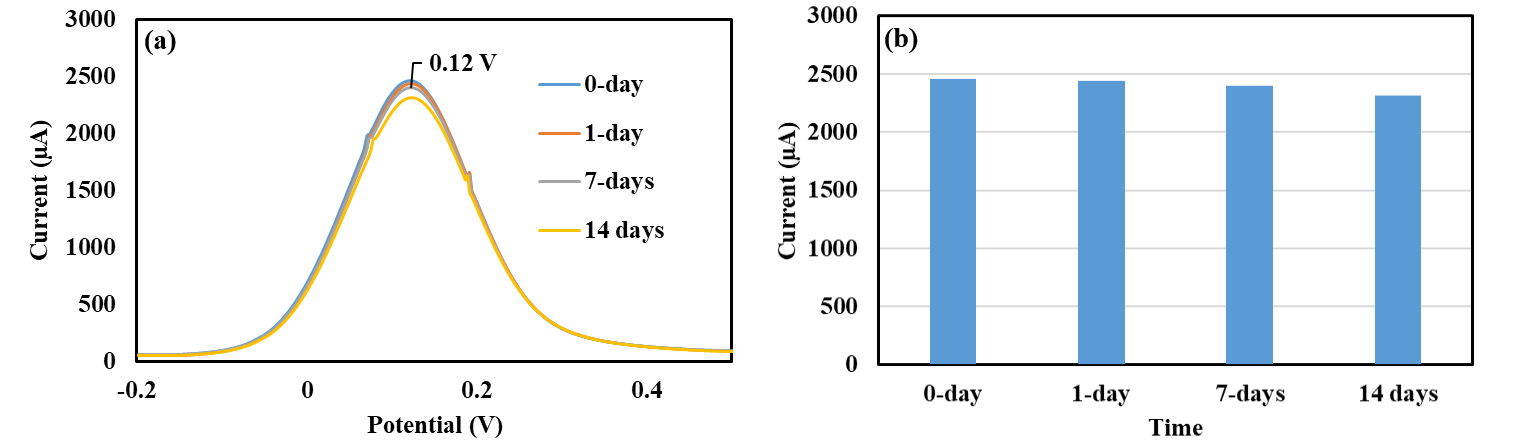


Fig. S8. Change in SWV current of the electrochemical immunosensor at 0, 1, 7, and 14 days.


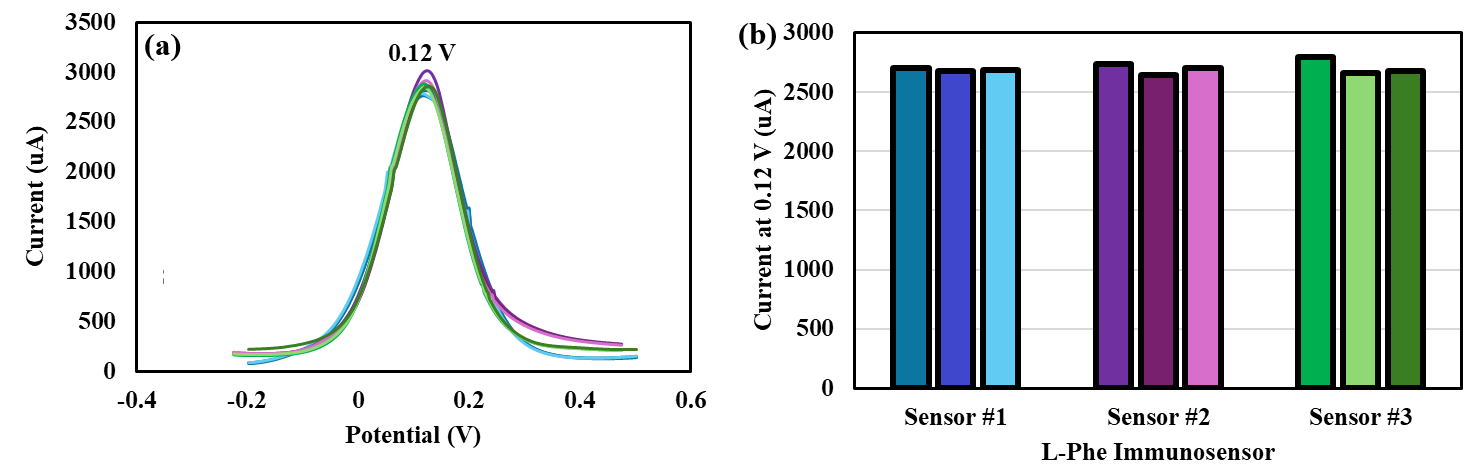


Fig S9 Reproducibility of the L-Phe immunosensor (a) SWV measurements and (b) current at 0.12 V.


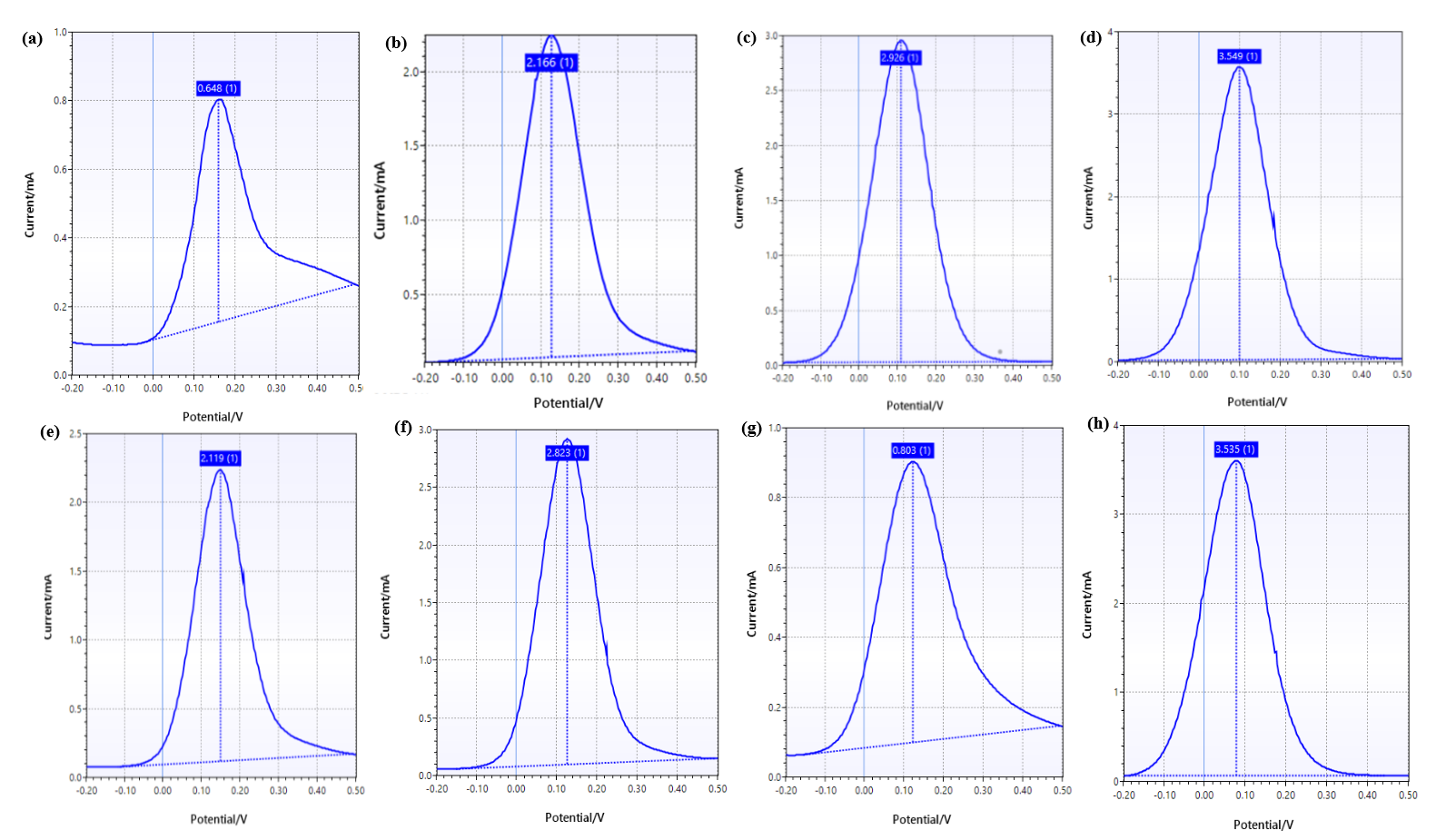


Fig.S10. SWV measurements of ERNDIM DBS samples (a) DBS 1, (b) DBS 3, (c) DBS 5, (d) DBS 7, (e) DBS 9, (f) DBS 11, (g) DBS 13, (h) DBS 15


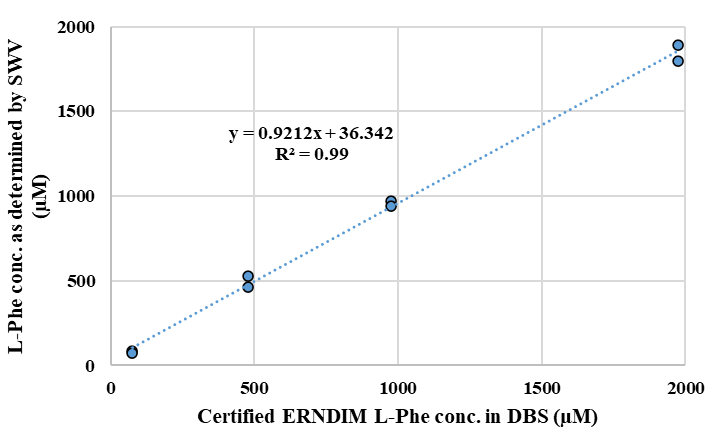


Fig.S11. Linearity between the L-Phe concentration determined by the new immunosensor using SWV measurements and the certified concentrations of L-Phe in the DBS samples provided by ERNDIM.

Table S2: Z-Score comparison between SWV method and Lab1 and Lab 2.

| **DBS No.** | **Z-Score**  **(this work)** | **Lab 1 (µM)** | **Z-score** | **Lab 2 (µM)** | **Z-score** | **Peer Mean** | **Peer Median** | **Peer SD (MS)** |
| --- | --- | --- | --- | --- | --- | --- | --- | --- |
| **1** | 0.20 | 2006.3 | 0.54 | 1545.0 | -0.85 | 1822 | 1837 | 343 |
| **3** | 0.02 | 973.5 | 0.31 | 780.9 | -1.30 | 937 | 936 | 119 |
| **5** | -0.28 | 444.2 | -0.62 | 427.9 | -0.89 | 482 | 482 | 60.8 |
| **7** | -2.75 | 103.7 | -0.42 | 98.9 | -0.88 | 109 | 110 | 12.6 |
| **9** | 0.41 | 887.3 | -0.28 | 568.0 | -3.03 | 920 | 920 | 116 |
| **11** | 0.99 | 438.1 | -0.86 | 440.0 | -0.84 | 481 | 482 | 49.7 |
| **13** | 1.5 | 1606.5 | -0.69 | 1495.6 | -1.15 | 1762 | 1754 | 224 |
| **15** | -1.93 | 95.3 | -1.01 | 89.4 | -1.52 | 109 | 110 | 13.5 |

Table S3: Precision of the paired samples.

| **Samples** | **SD** | **CV%** |
| --- | --- | --- |
| 7, 15 | 6.02 | 7.67 |
| 5, 11 | 37.77 | 7.50 |
| 3, 9 | 20.00 | 2.10 |
| 1, 13 | 66.93 | 3.63 |


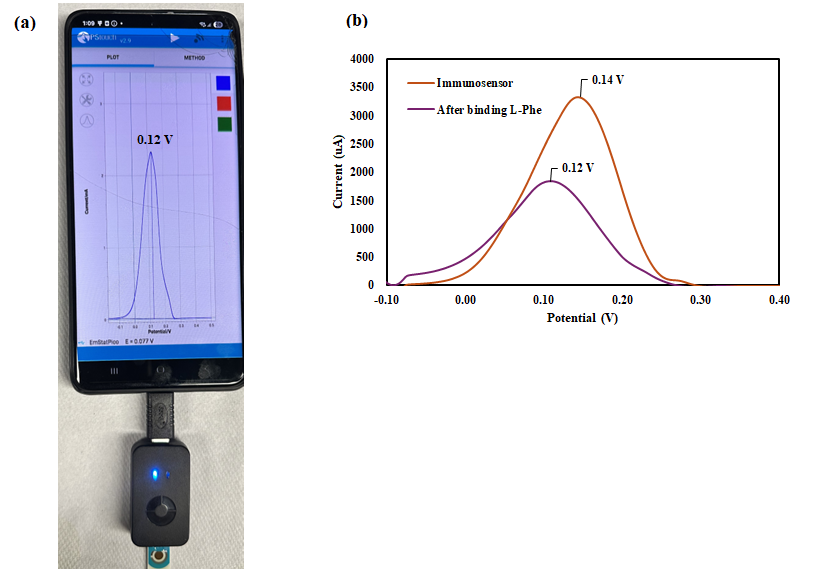


Fig.S12. (a) Detection of L-Phe in DBS sample by an ultra-compact potentiostat and a smartphone, (b) SWV of the new electrochemical immunosensor before and after L-Phe binding from DBS sample. The measurements were caried out using the ultra-compact potentiostat and a smartphone.

References

1. Rule KL, Vikesland PJ. (2009) Surface-enhanced resonance raman spectroscopy for the rapid detection of cryptosporidium parvum and giardia lamblia. Environmental science & technology 43:4:1147-52.

2. Halvorson RA, Leng W, Vikesland PJ. (2011) Differentiation of microcystin, nodularin, and their component amino acids by drop-coating deposition raman spectroscopy. Analytical chemistry 83:24:9273-80.

3. Zhu G, Zhu X, Fan Q, Wan X. (2011) Raman spectra of amino acids and their aqueous solutions. Spectrochimica Acta Part A: Molecular and Biomolecular Spectroscopy 78:3:1187-95.

4. Rygula A, Majzner K, Marzec KM, Kaczor A, Pilarczyk M, Baranska M. (2013) Raman spectroscopy of proteins: A review. Journal of Raman Spectroscopy 44:8:1061-76.

5. Stewart S, Fredericks P. (1999) Surface-enhanced raman spectroscopy of amino acids adsorbed on an electrochemically prepared silver surface. Spectrochimica Acta Part A: Molecular and Biomolecular Spectroscopy 55:7-8:1641-60.

6. Nihal S, Sarfo D, Zhang X, Tesfamichael T, Karunathilaka N, Punyadeera C, et al. (2024) Paper electrochemical immunosensor for the rapid screening of galectin-3 patients with heart failure. Talanta 274:126012.

7. Podstawka E, Ozaki Y, Proniewicz LM. (2005) Part iii: Surface-enhanced raman scattering of amino acids and their homodipeptide monolayers deposited onto colloidal gold surface. Applied spectroscopy 59:12:1516-26.

8. Kho KW, Dinish U, Kumar A, Olivo M. (2012) Frequency shifts in sers for biosensing. ACS nano 6:6:4892-902.
